# Fusobacterium necrophorum Septicemia Leading to Lemierre’s Syndrome in an Immunocompetent Individual: A Case Report

**DOI:** 10.7759/cureus.7443

**Published:** 2020-03-28

**Authors:** Balarama K Surapaneni, Hanad Omar, Michele M Iguina, Manuel Suarez

**Affiliations:** 1 Internal Medicine, Aventura Hospital and Medical Center, Aventura, USA

**Keywords:** fusobacterium necrophorum, lemierre's syndrome, thrombophlebitis, immunocompetent

## Abstract

Lemierre syndrome is a life-threatening condition associated with infection by obligate anaerobes residing in oropharyngeal mucosa. The most common organism responsible is Fusobacterium necrophorum. We report a case in a 69-year-old gentleman. The man with past medical history of hypertension, anxiety and chronic alcohol abuse was brought in by his family for altered mental status and fever. He had a complicated stay with septic shock on multiple pressors, his blood cultures grew Fusobacterium necrophorum and neck ultrasound showed acute thrombus of the right internal jugular vein (IJV). The patient had received intravenous antibiotics throughout stay but had poor prognosis and eventually expired after a complicated hospital stay.

Lemierre syndrome is a rare syndrome usually associated with an acute oropharyngeal infection due to anaerobic bacteria leading to secondary septic thrombophlebitis of the internal jugular vein. The characteristic clinical picture noticed is a hematogenous progression to distant septic emboli. It is a life-threatening condition and a prompt diagnosis is critical for preventing fatal consequences.

The purpose of this case report is to increase awareness about this clinical condition among medical professionals.

## Introduction

Lemierre’s syndrome (LS) is typically caused by the obligate anaerobe Fusobacterium necrophorum, which resides in the oropharyngeal mucosa. We report a 69-year-old gentleman who came to hospital altered and had a complicated hospital stay and over the course of the admission found to have Fusobacterium necrophorum septicemia and right internal jugular vein (IJV) thrombosis.

## Case presentation

**Figure 1:**
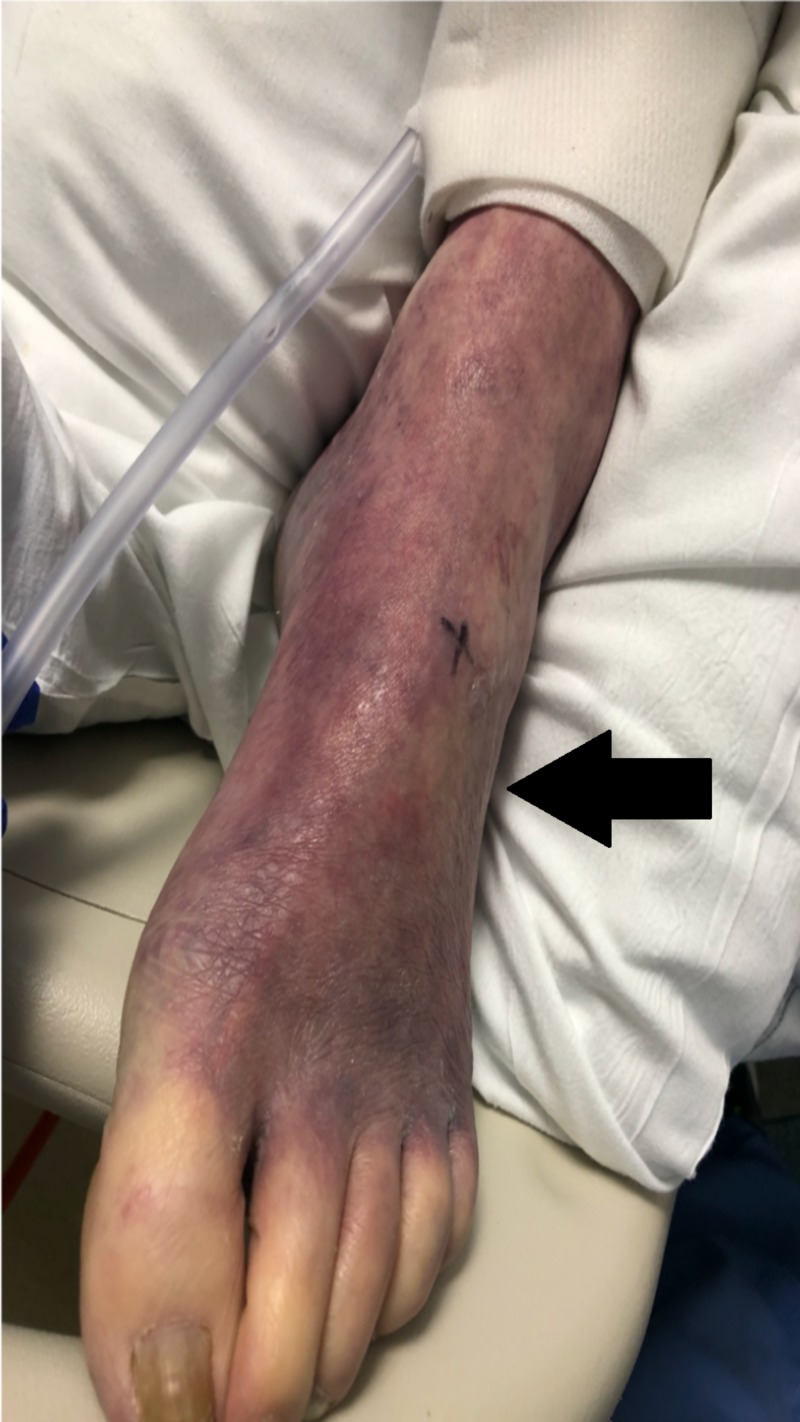
Peripheral cyanosis - Ischemic leg Ischemia of patient’s leg suspect secondary to ischemic emboli.

**Figure 2:**
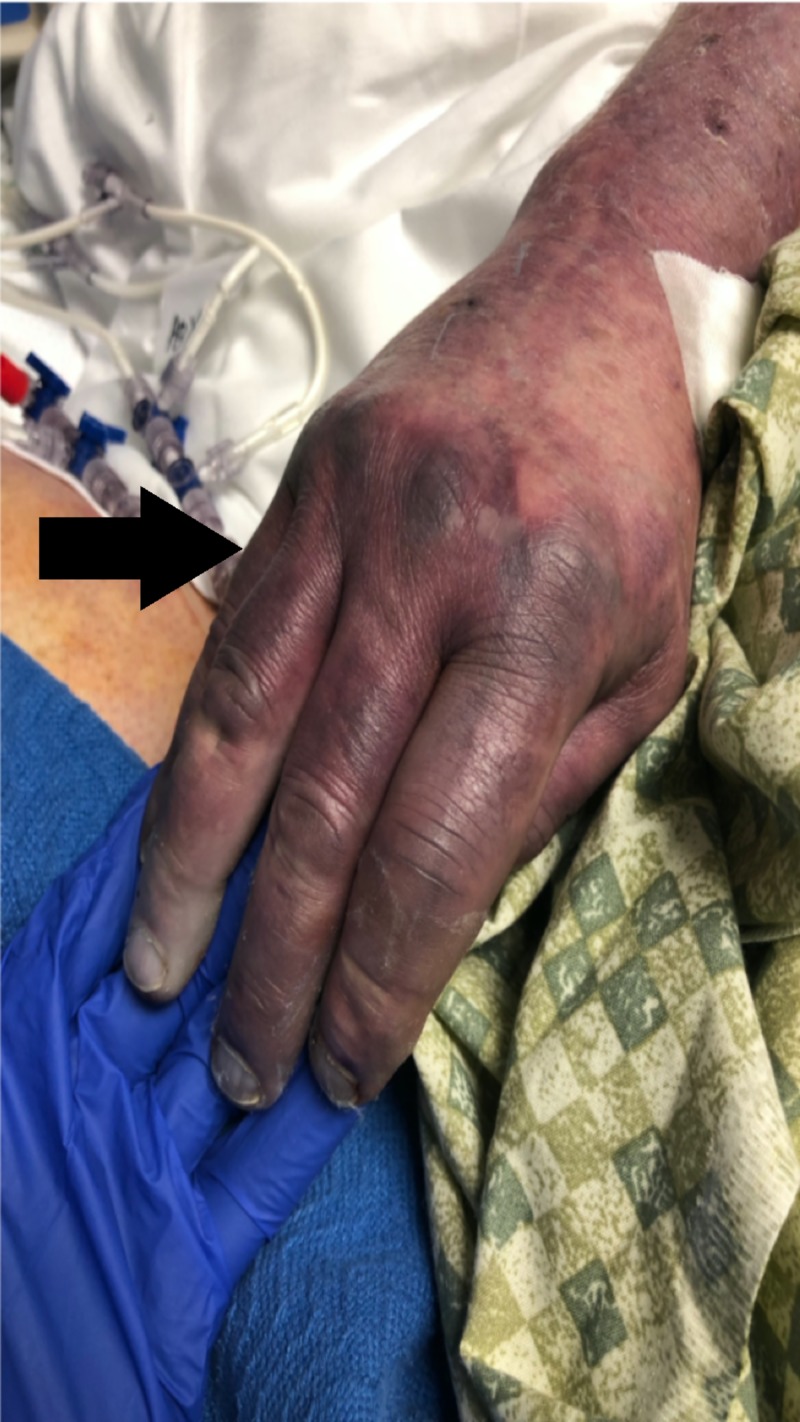
Peripheral cyanosis - Ischemic hand Ischemia of patient’s hand suspect secondary to septic emboli.

**Figure 3:**
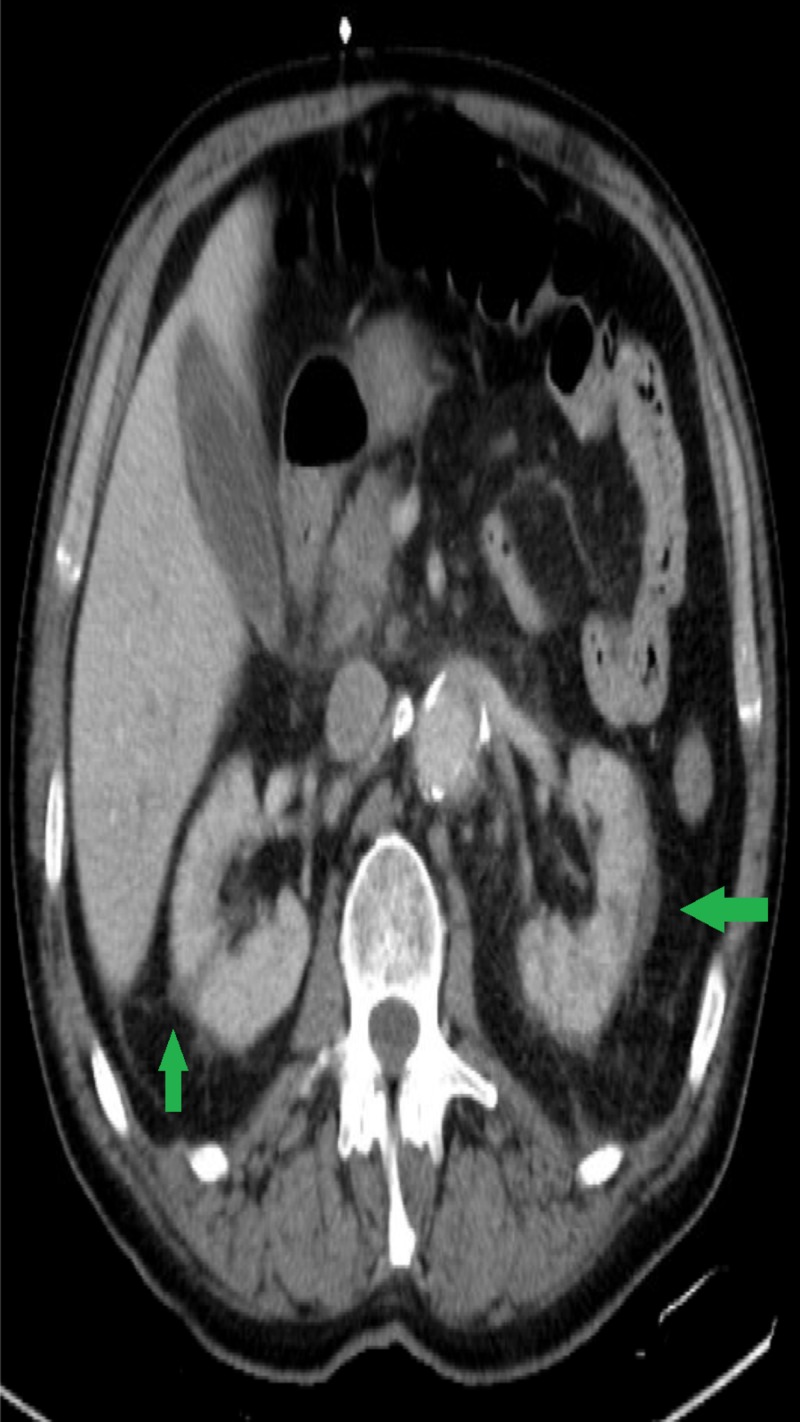
Renal infarcts Bilateral wedge-shaped renal infarcts suspect secondary to emboli.

**Figure 4:**
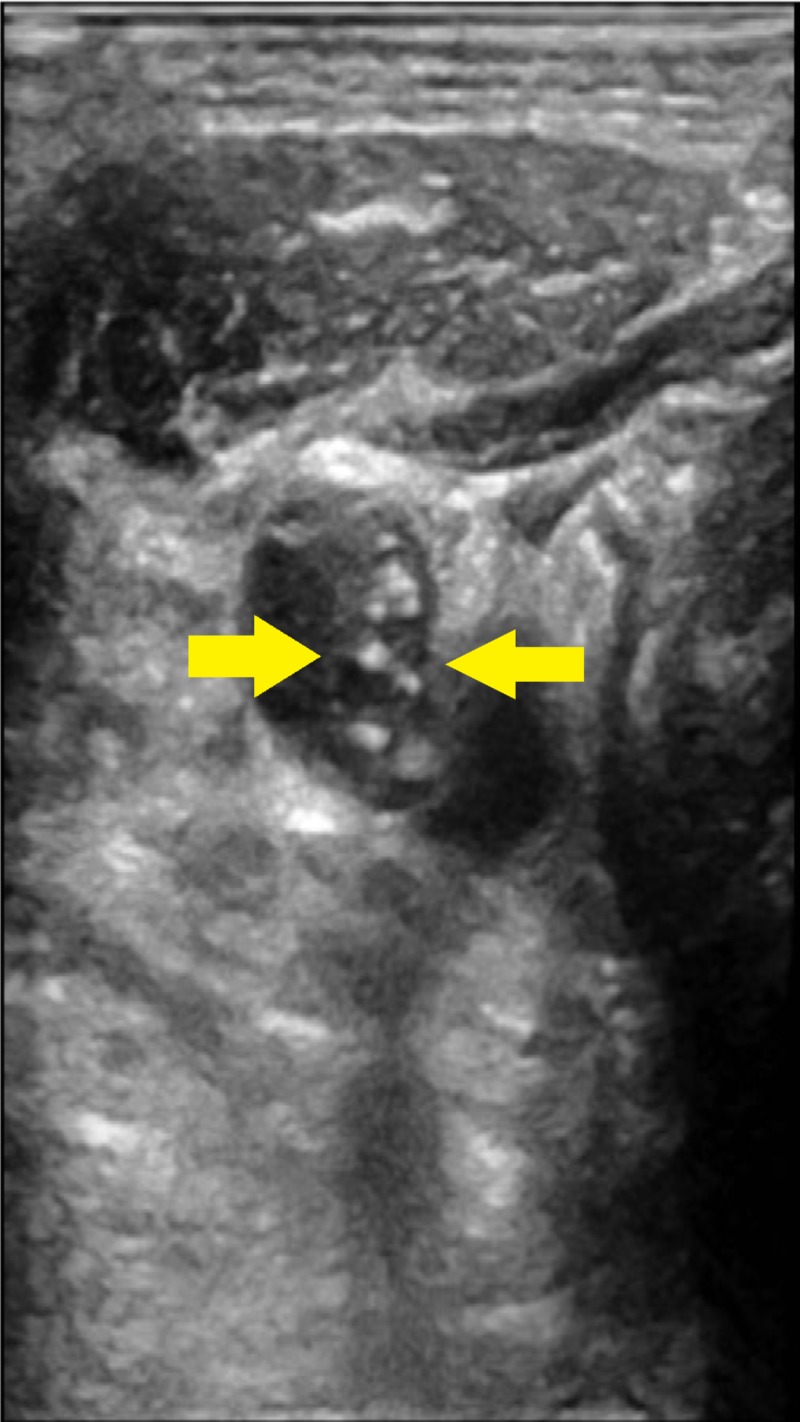
Right internal jugular vein (IJV) thrombus Thrombus formation in the right internal jugular vein.

A 69-year-old surgeon with a past medical history of hypertension, anxiety and chronic alcohol abuse was brought in by his family for altered mental status and fever. His initial presentation was notable for a temperature of 106.2 F, central and peripheral cyanosis (Figures 1, 2), profound hypoxia, scattered purpura and extremely poor dentition. He was intubated for acute hypoxic respiratory failure, and treated in the emergency room with broad spectrum antibiotics including meningitis coverage, dantrolene for possible neuroleptic malignant syndrome and methylene blue for methemoglobinemia on his arterial blood gas. The patient was also started on three vasopressors for hemodynamic support. On presentation his CT brain was unremarkable, his CT abdomen/pelvis was notable for a 7.2-cm infra-renal abdominal aortic aneurysm and bilateral renal wedge shaped infarcts (Figure 3). The patient’s condition continued to worsen and he developed multi-organ failure, rhabdomyolysis and a non-ST-segment elevation myocardial infarction. On the third day of his hospitalization, his blood cultures grew Fusobacterium necrophorum. Neck ultrasound was ordered which showed acute thrombus of the right IJV (Figure 4).

The patient was not waking up off sedation and believed to have suffered an anoxic brain injury. All four extremities were also blue and cold, without pulses by Doppler. The patient had very clear advanced directives that if he were to ever be critically ill with no signs of meaningful recovery that he would not want to be on life support and so the family decided to withdraw care and the patient expired.

## Discussion

Lemierre’s syndrome (LS) is a syndrome that is characterized by anaerobic bacteremia and thrombophlebitis of the internal jugular vein (IJV). It is a rare syndrome, typically found in immunocompetent children and adults and carries a very high mortality [1]. Since the advent of antibiotics in the 1960s, the incidence of this disease has dramatically decreased. Attributed primarily due to use of penicillin for oropharyngeal infection was a significant cause for the dramatic decline of this condition [2]. Risk factors in adults include recurrent pharyngitis/tonsillitis or persistent fevers or history of sinusitis or dental procedures [3]. The time difference between oropharyngeal infection to onset of septicemia is usually one week in most of infections. Our patient had poor dentition on examination and required multiple pressors for hemodynamic support for septic shock.

Hallmark for diagnosis is presence of thrombophlebitis of the IJV with blood cultures usually growing Fusobacterium necrophorum [4]. Other organisms isolated in Lemierre disease include Streptococcus species, Bacteroides species, Peptostreptococcus species and Eikenella corrodens [5]. Lungs are the most common site for septic emboli. Sinave et al. reported pleuropulmonary septic emboli in 97% of the cases [5]. Other sites involved include joints, bone, meninges, and liver in the literature [6].

Treatment is usually antibiotic coverage targeting anaerobic flora in patients. Patients on antibiotics should be closely observed and monitored for signs of continued sepsis, propagation of thrombus and septic emboli formation [5]. Anticoagulation therapy is usually controversial as efficacy reported in small case series as there is no consensus if the therapy is helpful in patients with internal jugular vein thrombosis [3]. Most of the patients who received anticoagulation were individualised and no controlled studies exist. It can be used as a last resort in patients not responding to antibiotics or progression of thrombosis or retrograde cavernous sinus thrombosis. Duration of anticoagulation is a matter of debate with recommendations varying from weeks to months depending on location and complexity of thrombosis [7-9]. Based on a systematic review of 84 observational studies evaluating prognosis of patients with Lemierre syndrome 58% of patients required intensive care in analysis of 84 studies with 114 patients with Lemierre syndrome [10].

## Conclusions

This case illustrates the severity of this rare but life-threatening syndrome. Association of thrombophlebitis of the internal jugular venous system, its branches, and septic embolization often leads to fulminant organ failure. There are multiple cases reported in the literature about Fusobacterium necrophorum septicemia leading to Lemierre’s syndrome. It is critical to identify it early. Prompt diagnosis and antibiotic coverage that includes anaerobic organisms may lead to better outcomes, however in this patient’s case the profound septicemia and metastatic infection was likely too advanced to overcome. We hereby report and discuss the pathogenesis, diagnosis, and treatment strategies for this entity in regard to their clinical presentation and outcome.
